# Characterizing preclinical sub‐phenotypic models of acute respiratory distress syndrome: An experimental ovine study

**DOI:** 10.14814/phy2.15048

**Published:** 2021-10-07

**Authors:** Jonathan E. Millar, Karin Wildi, Nicole Bartnikowski, Mahe Bouquet, Kieran Hyslop, Margaret R. Passmore, Katrina K. Ki, Louise E. See Hoe, Nchafatso G. Obonyo, Lucile Neyton, Sanne Pedersen, Sacha Rozencwajg, J. Kenneth Baillie, Gianluigi Li Bassi, Jacky Y. Suen, Daniel F. McAuley, John F. Fraser

**Affiliations:** ^1^ Critical Care Research Group The Prince Charles Hospital Brisbane Australia; ^2^ Faculty of Medicine University of Queensland Brisbane Australia; ^3^ Roslin Institute University of Edinburgh Edinburgh UK; ^4^ Cardiovascular Research Institute Basel Basel Switzerland; ^5^ Institute of Health and Biomedical Innovation Queensland University of Technology Australia; ^6^ Wellcome Trust Centre for Global Health Research Imperial College London UK; ^7^ Sorbonne Universités UPMC Université Paris 06 INSERM UMRS‐1166 ICAN Institute of Cardiometabolism and Nutrition, Medical ICU Pitié‐Salpêtrière University Hospital Paris France; ^8^ Wellcome‐Wolfson Institute for Experimental Medicine Queen’s University Belfast Belfast UK

**Keywords:** acute respiratory distress syndrome, animal, models, phenotype

## Abstract

The acute respiratory distress syndrome (ARDS) describes a heterogenous population of patients with acute severe respiratory failure. However, contemporary advances have begun to identify distinct sub‐phenotypes that exist within its broader envelope. These sub‐phenotypes have varied outcomes and respond differently to several previously studied interventions. A more precise understanding of their pathobiology and an ability to prospectively identify them, may allow for the development of precision therapies in ARDS. Historically, animal models have played a key role in translational research, although few studies have so far assessed either the ability of animal models to replicate these sub‐phenotypes or investigated the presence of sub‐phenotypes within animal models. Here, in three ovine models of ARDS, using combinations of oleic acid and intravenous, or intratracheal lipopolysaccharide, we investigated the presence of sub‐phenotypes which qualitatively resemble those found in clinical cohorts. Principal Component Analysis and partitional clustering identified two clusters, differentiated by markers of shock, inflammation, and lung injury. This study provides a first exploration of ARDS phenotypes in preclinical models and suggests a methodology for investigating this phenomenon in future studies.

## Graphical abstract

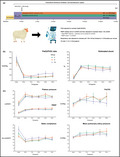

## 1 INTRODUCTION

It is increasingly understood that the acute respiratory distress syndrome (ARDS) describes a clinically and immunologically heterogenous population (Sinha & Calfee, 2019). Heterogeneity among patients with ARDS has been proffered as an explanation for consistently negative trials of pharmacological treatments. Contemporary advances in phenotyping, using unsupervised machine learning techniques, have identified novel sub‐phenotypes in clinical trial cohorts (Calfee et al., 2014). These phenotypes have discrepant outcomes, and importantly, appear to respond differently to several interventions (Calfee et al., 2018). An ability to prospectively identify sub‐phenotype membership in patients with ARDS opens the possibility of delivering personalized treatments.

Historically, animal models of ARDS have played an important role in biological discovery and in therapeutic translation (Yehya, 2019). Numerous models of ARDS have been developed in both large and small animals. However, an animal model that fully recapitulates the clinical pathobiology of ARDS is not available. This has contributed to the gap between results generated from preclinical models and those obtained in subsequent clinical trials. As our knowledge of clinical sub‐phenotypes grows, a new question arises for those modelling ARDS in animals; how well does an animal model reflect the pathobiology of a specific clinical sub‐phenotype? To answer this question several preliminary facts need to be elucidated. Do existing preclinical models of ARDS more closely resemble one phenotype or another? And, do animals with experimental ARDS exhibit phenotypes given a common method of injury?

Thus, we sought to develop an approach to these problems by testing three models of ARDS in sheep. Using a combination of dimensionality reduction and partitional clustering, we investigated the presence of sub‐phenotypes, arising dependent or independent of the means of injury. Previously, others have pursued a related approach to identify sub‐phenotypes in a murine model of sepsis (Seymour, Kerti, et al., 2019). Similarly, we aimed to undertake a preliminary exploration of sub‐phenotypes arising in preclinical models of ARDS, and to propose a methodology for investigating these phenomena in animal models.

## 2 MATERIALS AND METHODS

### 2.1 Study design

**FIGURE 1:**
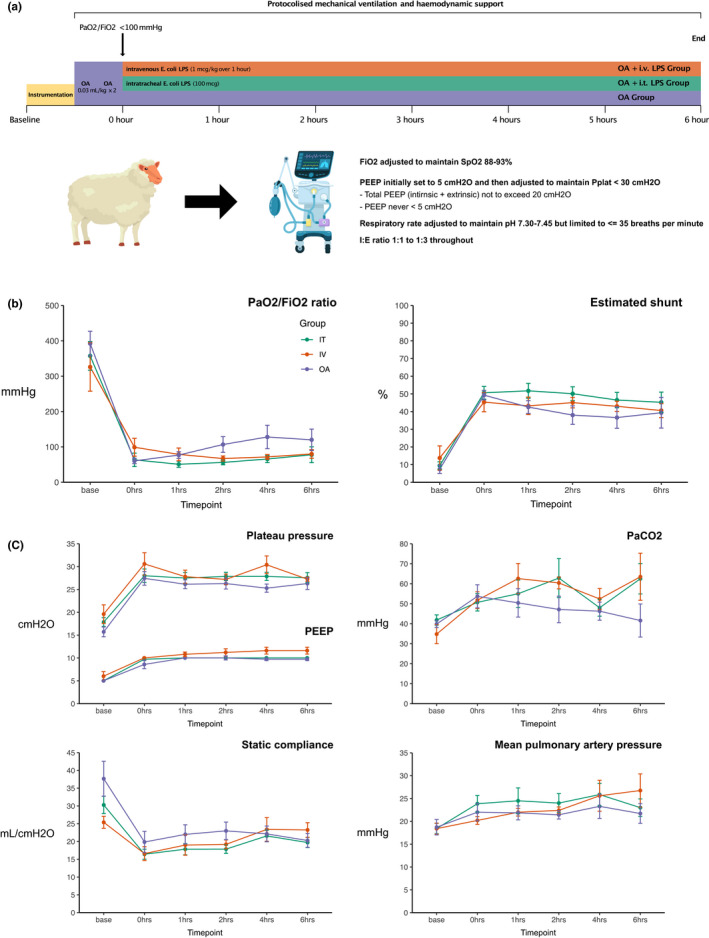
Study overview, measures of gas exchange, and respiratory mechanics. (a) Schematic overview of study design. (b) Measures of gas exchange. (c) Measures of respiratory mechanics. Data are presented as mean and 95% confidence intervals

Ethical approval for this study was obtained from University Animal Ethics Committees (QUT1600001108, UQPCH/483/17). The study was conducted in accordance with the Australian Code of Practice for the Care and Use of Animals for Scientific Purposes (Council NHMR, 2013), and reported in compliance with the ARRIVE guidelines (Percie du Sert et al., 2019). Detailed methods are provided in an online supplement. A diagrammatic summary of the study is presented in Figure 1.

### 2.2 Animal model

Nineteen healthy Border Leicester Cross ewes, aged between 1 and 3 years and weighing 52 kg (47–54), were randomly assigned to one of three groups; injury by intravenous infusion of oleic acid (OA, n = 7), by OA and intratracheal *E*. *coli* lipopolysaccharide (IT, n = 7), or by OA and intravenous *E*. *coli* lipopolysaccharide (IV, n = 5).

Briefly, animals were anesthetized with ketamine, midazolam, and fentanyl. Continuous neuromuscular blockade was maintained by infusion of vecuronium. After induction, animals were tracheostomized and ventilated using a low tidal volume strategy. After instrumentation, acute lung injury was induced by infusion of OA (0.06 ml/kg; O1008, Sigma‐Aldrich, Castle Hill, NSW, Australia), with or without, intratracheal *E*. *coli* LPS (100 µg; O55:B5, Sigma‐Aldrich, Castle Hill, NSW, Australia) or intravenous *E*. *coli* LPS (1 μg/kg infused over 1 h; O55:B5, Sigma‐Aldrich, Castle Hill, NSW, Australia). Once a PaO_2_/FiO_2_ ratio <100 mmHg (PEEP ≥5 cmH_2_O) was achieved (0 h), animals received protocolized intensive care for the duration of the study. At 6 h, animals were euthanized.

### 2.3 Blood and cytokine analysis

Blood samples were analyzed by and independent veterinary laboratory (IDEXX Laboratories, Brisbane, Australia) to clinical standards. Routine biochemical, hematological, and coagulation tests were performed. The concentration of interleukin‐6 (IL‐6), IL‐8, IL‐1β, and IL‐10 was measured in blood and in bronchoalveolar lavage (BAL) fluid. Our development of ovine‐specific ELISA assays has been described before (Bouquet et al., 2020). Detailed methods are provided in an online supplement.

### 2.4 Statistical analysis

Data are expressed as median (IQR). Analysis was undertaken in R 4.0.3 (R Core Team, Vienna, Austria, 2020). The dataset for this study, along with reproducible code and supplementary methods and results, is available at http://doi.org/10.5281/zenodo.4677513. Longitudinal data were analyzed by fitting linear mixed models, using the R package *lme4*. Non‐longitudinal data were compared with one‐way ANOVA. Where a significant interaction was observed, post hoc comparisons were made using Tukey's test, using the R package *rstatix*. Correction for multiple comparisons was made using the Benjamini–Hochberg method. Frequency data were compared using the Chi‐squared test. Co‐linearity was assessed by calculating the Spearman correlation coefficient for each pair of variables, using the R package *corrplot*. Principal component analysis (PCA) was performed to reduce the dataset to a smaller number of principal components (PCs), using the R package *FactoMineR* (Husson et al., 2017). Beforehand, missing data were imputed using a random forest approach with predictive mean matching (using the R package *missRanger*) and the dataset was z‐score normalized by subtracting the variable mean and dividing by the variable standard deviation (Josse & Husson, 2012). After examination of the scree plot, principal components sufficient to explain >75% of the total variance were retained. Partitioning around medoids (PAM) clustering was performed, after PCA, on the imputed dataset, using the R package *cluster*. A Euclidean distance measure was employed. The optimal number of clusters to specify was derived from a “majority” assessment of 26 measures, using the *NbClust* package. In event of a tie a parsimonious solution was preferred. To assess the stability of clusters, we employed a nonparametric bootstrap‐based strategy using the R package *fpc*. This generated 1000 new datasets by randomly drawing samples from the initial dataset with replacement and applying the same clustering technique to each. Clustering results were then compared for each cluster identified in the primary analysis and the most similar cluster identified for each random re‐sampling. A mean value for the Jaccard coefficient, for the sum of the comparisons, was generated for each cluster. Z‐scores for each variable, by cluster, were descriptively compared with clusters derived from a previously published latent class analysis of the ARMA study (Calfee et al., 2014), obtained using a digital ruler. Statistical significance was assumed if *p *< 0.05.

## 3 RESULTS

Baseline characteristics at injury (0 h) are summarized in Table 1 and in Table E1 (Supplementary results available at http://doi.org/10.5281/zenodo.4677513). All animals completed the study protocol and were euthanized at 6 h (Table E2). There were some missing data in our study (Table E3) and prior to analysis these data were imputed using a random forest method.

**TABLE 1:** Physiological characteristics at 0 hours (injury). Data are presented as median (IQR)

	Overall (n=19)	OA (n=7)	IT (n=7)	IV (n=5)
Weight (kg)	52 (47–54)	55 (53–57)	47 (46–51)	52 (46–52)
PEEP (cmH_2_O)	10 (10–10)	10 (7.5–10)	10 (10–10)	10 (10–10)
Plateau pressure (cmH_2_O)	27 (26–30)	26 (26–27)	27 (26–29)	29 (28–34)
Static compliance (mL/cmH_2_O)	16 (14–22)	21 (16–25)	16 (14–18)	16 (14–16)
PaO_2_/FiO_2_ (mmHg)	52 (47–90)	52 (49–69)	47 (46–51)	100 (55–133)
Effective shunt (%)	49 (43–54)	49 (44–54)	50 (48–54)	45 (37–52)
PaCO_2_ (mmHg)	49 (46–57)	47 (46–53)	52 (46–57)	49 (48–53)
pH	7.31 (7.28–7.35)	7.31 (7.25–7.32)	7.33 (7.3–7.38)	7.32 (7.31–7.37)
Bicarbonate (mmol/L)	23.8 (22.3–24.9)	22.4 (21.9–23.3)	24.3 (23.9–24.9)	23.4 (22.6–25.4)
Base excess (mmol/L)	−0.6 (−2.5 to 1.1)	−2.5 (−3 to −2.4)	1 (0–1.8)	0.8 (−1.4 to 2.6)
Heart rate (bpm)	112 (102–134)	121 (96–134)	111 (100–132)	112 (106–117)
Mean arterial pressure (mmHg)	96 (85–109)	104 (96–111)	80 (76–92)	108 (99–115)
Mean pulmonary arterial pressure (mmHg)	21 (19–25)	22 (19–26)	23 (20–27)	20 (19–21)
Central venous pressure (mmHg)	12 (9–13)	13 (12–15)	11 (9–13)	11 (3–19)

Oleic acid, with or without LPS, induces acute severe respiratory failure. However, LPS prolongs or prevents improvements in oxygenation in the first 6 h

The infusion of OA produced severe lung injury (PaO_2_/FiO_2_ <100 mmHg). However, oxygenation began to recover within 1 h (Figure 1). LPS consistently maintained PaO_2_/FiO_2_ within the range of severe ARDS. Differences in oxygenation between groups were not associated with differences in respiratory system mechanics (Figure 1).

### 3.1 The addition of LPS increases the plasma concentration of interleukin‐6

Both intravenous and intratracheal LPS increased the plasma concentration of IL‐6 (group:time, *p* <0.001) when compared to OA alone (Figure E1 and Tables E4 and E5). The use of intravenous LPS was associated with non‐sustained increases in the plasma concentration of IL‐8 (group:time, *p *< 0.001). Similarly, the use of intravenous LPS resulted in increase in plasma IL‐10 (group:time, *p *< 0.001). All three injury methods were associated with low plasma levels of IL‐1β. Cytokine concentrations in bronchoalveolar lavage (BAL) fluid were less distinct between groups (Figure E1 and Tables E4 and E5). Animals injured with intravenous LPS, as compared to OA alone, had higher white cell and neutrophil counts at 6 h (*p *= 0.049 and 0.034, respectively).

### 3.2 In the first 6 hours, intravenous LPS induces more severe shock in comparison to intratracheal LPS or OA alone

All pulmonary injury methods produced shock requiring vasopressor support (Figure E2). While reductions in MAP from baseline to 6 h were greater in animals receiving LPS, these differences were not statistically significant. Similarly, there were no significant differences in heart rate or CVP, between groups (Figure E2). Cumulative fluid balance was greatest in OA animals, and significantly different when compared to sheep given LPS (Figure E2). Cumulative urine output did not differ between groups (*p *= 0.109).

Hematological and biochemical parameters, at 6 h, are presented in Table E2. Following correction for multiple comparisons, there were no significant differences between groups in indices of renal function.

### 3.3 Two sub‐phenotypes are identifiable and are associated with the method of injury

**FIGURE 2:**
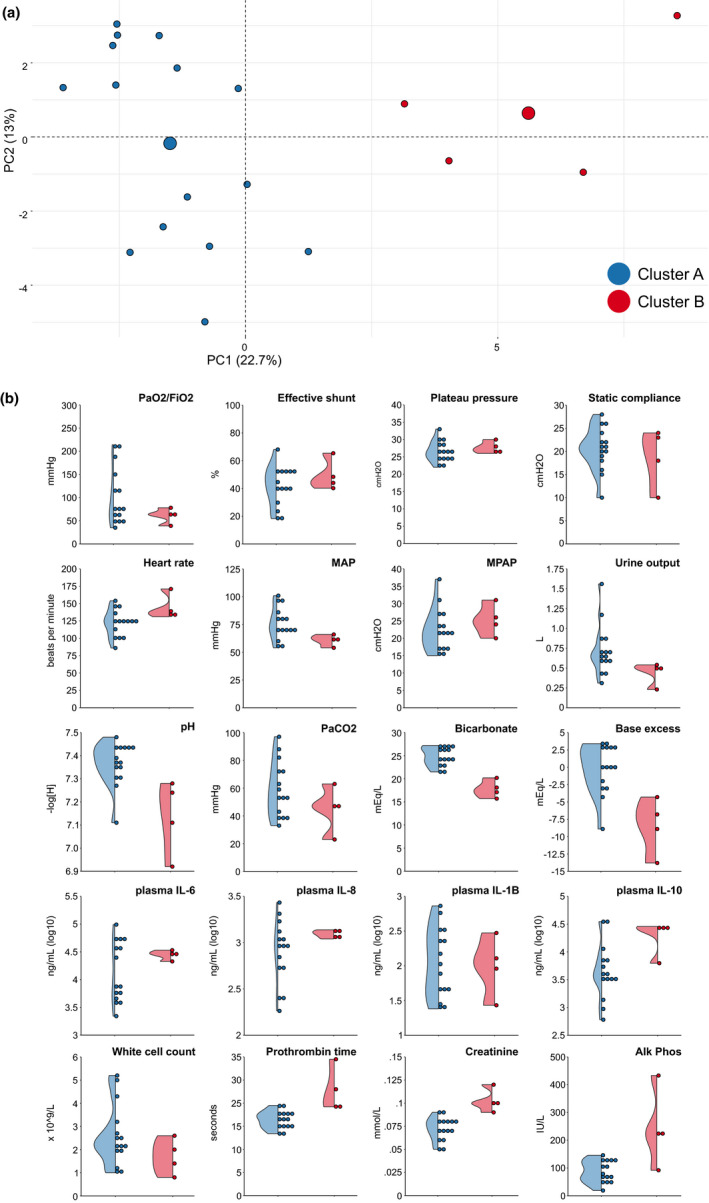
Partitional clustering and sub‐phenotypes. (a) Clustering results projected on PCs 1 and 2 of the PCA. B. Half‐dot, half‐violin plots of key variables stratified by cluster membership

A priori assessment of the data suggested that two clusters were the optimal partition of our data (Table E6). The distribution of the clusters in two‐dimensional space is shown in Figure 2. Animals in cluster B (n = 4) tended to have a lower MAP, lower urine output, were more acidotic, coagulopathic, and had higher plasma levels of IL‐6, IL‐8, and IL‐10 (Figure 2). Proportionally, more animals injured with intravenous LPS were found in Cluster B (*p *= 0.036). All animals injured with IT LPS were assigned to Cluster A. The clusterwise stability was good (mean Jaccard similarities, 0.85 Cluster A, 0.71 Cluster B).

### 3.4 Differences in variables describing the severity of shock and of lung injury explain the majority of variance between animals

**FIGURE 3:**
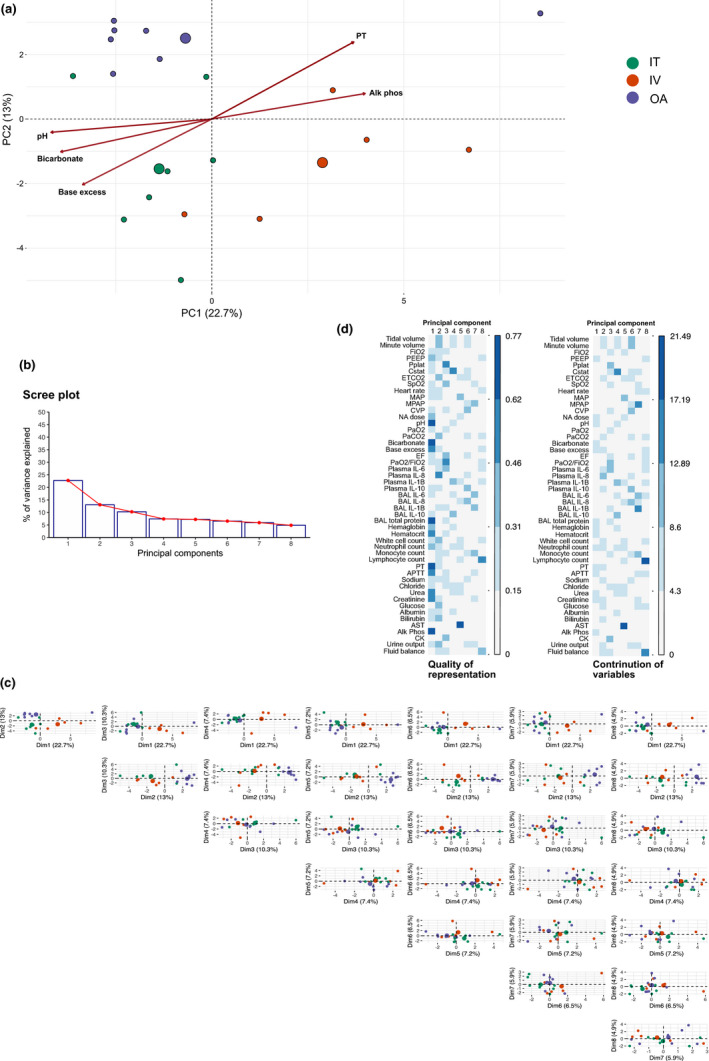
Principal component analysis (PCA). (a) Biplot of principal components (PCs) 1 and 2. The top five variables in PC 1 are shown. Large dots represent the group mean. (b) Scree plot of first eight PCs. (c) Pairs plot of PCA projections for first eight PCs. (d) Contribution and quality of representation of variables to PCs. The quality of representation (cos2) sums to one for each variable across all PCs. The contribution of variables to variance in a PC are expressed as percentages

**FIGURE 4:**
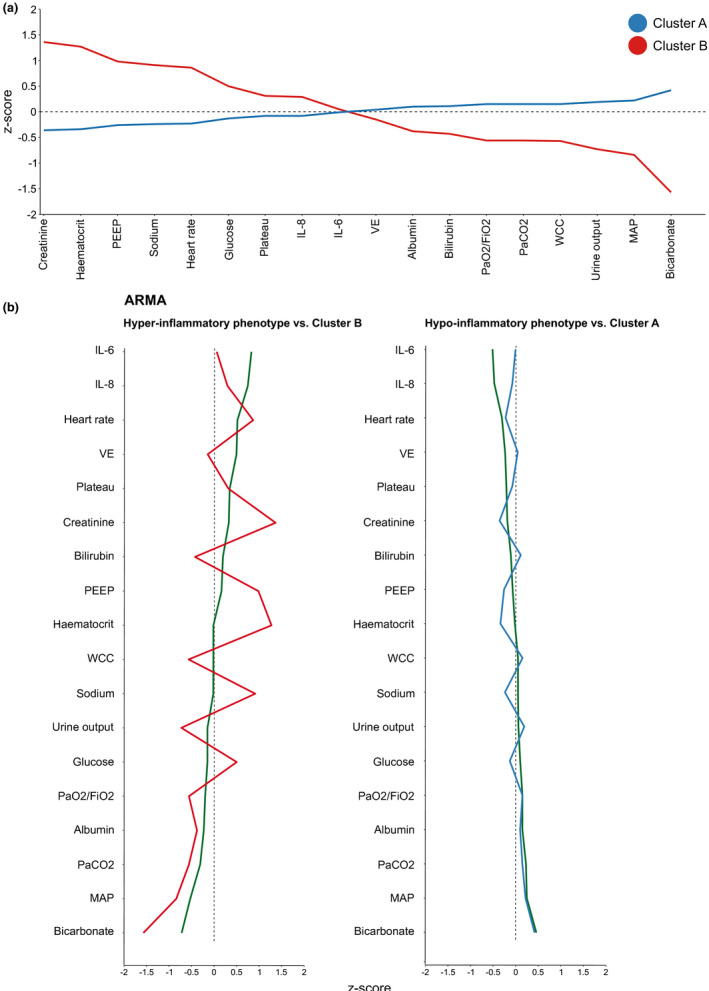
Preclinical clusters and clinical cohorts. (a) Z‐core plot of animal clusters for variables common with those in published clustering studies. VE, minute volume, WCC, white cell count, MAP, mean arterial pressure. (b) Preclinical cluster z‐score plots contrasted with clinical trial clustering sub‐phenotypes. Green line, ARMA trial sub‐phenotypes

There was a high degree of correlation between variables recorded in our study at 6 h (Figure E3). In an effort to reduce the dimensionality of the data and identify the most differentiating variables we performed principal component analysis (PCA). In total, eight principal components (PCs) explained >75% of the variance between animals, with the first 4 PCs explaining >50% (Figure 3). Markers of shock severity contributed heavily to PCs 1 and 2, while markers of lung injury defined PCs 4 and 5 (Figures 3 and 4 and Figure E4).

## 4 DISCUSSION

In a study combining three ovine models of ARDS, we provide a preliminary exploration of sub‐phenotypes occurring early in the course of illness. In doing so, we provide the first characterization of OA and LPS “double‐hit” models of ARDS in large animals. In this study, OA was capable of inducing severe acute respiratory failure, however oxygenation began to improve rapidly unless animals were additionally insulted with LPS. All three injury models produced an elevation in plateau airway pressure and a reduction in compliance. Likewise, all exhibited evidence of shock, requiring vasopressor support. Plasma levels of IL‐6 were higher in animals receiving intravenous LPS, however BAL concentrations remained unchanged. Decomposition of the dataset by PCA, revealed that the first 4 PCs explained 54% of the variance in the dataset, and may be characterized as representing the degree of shock/inflammation (PCs 1 and 2) and lung injury (PCs 3 and 4). Unsupervised clustering, using PAM, identified the presence of two sub‐phenotypes. These were primarily differentiated along PC1. Cluster membership was associated with method of injury with more animals belonging to the intravenous LPS group in cluster B. These data may have implications for translational research in ARDS. First, the presence of sub‐phenotypes in animal models opens the possibility of developing phenotype‐specific models. Second, unappreciated sub‐phenotypic variation in existing or future preclinical studies may result in differential treatment effects where interventions are being studied.

Several methods of inducing preclinical ARDS have been described in large animals (Yehya, 2019). In this study, we attempted to model the spectrum of host‐inflammatory responses, with OA at the “hypo‐inflammatory” end and OA and intravenous LPS at the “hyper‐inflammatory” extreme (intratracheal LPS being an intermediate). OA infusion is a classical model of ARDS, first described by David Ashbaugh and colleagues, in 1971 (King et al., 1971). The intravenous infusion of OA results in rapid‐onset lung injury, largely due to pulmonary endothelial damage and the formation of proteinaceous edema in alveoli (Wang et al., 2008). This is evident in our data, where OA infusion generated PaO_2_/FiO_2_ ratios <100 mmHg, within 30 min. OA has also been associated with the upregulation of inflammatory cytokines and chemokines, such as; IL‐6, IL‐8, TNF‐α, and matrix metalloproteinases (Ballard‐Croft et al., 2012; Gonçalves‐de‐Albuquerque et al., 2012). However, OA is not implicated in the activation of several signalling pathways known to be important in clinical ARDS, such as NF‐κB (Moine et al., 2000). In this study, animals receiving OA alone had consistently lower levels of pro‐inflammatory cytokines measured in plasma. These differences may also be accounted for by the fact that the injury caused by OA is predominantly pulmonary, as 85% of the free‐fatty acids from OA are retained in the lung (Gonçalves‐de‐Albuquerque et al., 2015). These features may restrict the ability of OA to accurately replicate the full pathobiology of ARDS, particularly ARDS of a non‐pulmonary etiology. The addition of LPS may address some of these limitations. As a common constituent of Gram‐negative bacterial cell walls, LPS participates in the pathology of causes of direct and indirect ARDS. The mechanisms of LPS‐mediated injury include; lung epithelial injury (involving NF‐κB induction), pulmonary endothelial damage, neutrophilic infiltration, and the activation of alveolar macrophages (Chen et al., 2010). The intravenous infusion of LPS is also associated with a systemic inflammatory response and the development of shock in preclinical models; as an alternative, by administering LPS via the intratracheal route, systemic effects may be limited, while inducing similar pathways within the lung (Wiener‐Kronish et al., 1991). In this study, the addition of LPS was associated with prolonged severe hypoxaemia, in contrast to OA alone.

An expanding number of studies has identified two consistent sub‐phenotypes of ARDS in clinical cohorts, which have been broadly characterized as hyper‐ and hypo‐inflammatory (Bos et al., 2017; Calfee et al., 2014, 2018; Famous et al., 2017; Sinha et al., 2018). These sub‐phenotypes have distinct outcomes and, in retrospective analysis, have been shown to respond differently to several interventions. Related sub‐phenotypes have also been identified in other conditions causing critical illness (Neyton et al., 2019; Seymour, Kennedy, et al., 2019; Vranas et al., 2017). However, the predominant clustering method employed in clinical cohorts, finite mixture modelling, is restricted in its applicability to preclinical studies, which are typically constrained to relatively small sample sizes (Dziak et al., 2014). Similarly, there are methodological challenges in assessing the similarity of these sub‐phenotypes between studies, exacerbated by a lack of understanding of the mechanisms underpinning their development and differences in the methods used to cluster groups. However, to date, no study has sought to identify these groupings by any means in animal models of ARDS.

In this study, partitional clustering, as opposed to model‐based clustering, was used to investigate the presence of sub‐phenotypes within the combined study. Partitional clustering is a common method to partition objects, in this case animals, into an optimal number of related clusters (Steinley, 2006). The aim is to maximize homogeneity within clusters while minimizing it between them. The algorithm is agnostic to the number of clusters, therefore an assessment of the optimal number of clusters was made before clustering was applied. This is unlike model‐based clustering where parametric techniques can be used to accept or refute the addition of classes versus a null model (usually starting with k = 1). However, we performed this assessment in a principled manner, accepting the consensus of 26 independent measures of cluster number. Here, two clusters provided the optimal solution. Mapping of individual animals on to the PCs derived by PCA allowed us to visualize the differences between clusters in a two‐dimensional space.

The sub‐phenotypes identified in this study share some qualitative similarities with those described in ARDS clinical cohorts. Cluster B animals in this study exhibited higher levels of plasma IL‐6, IL‐8, creatinine, and serum sodium, with elevated prothrombin times and heart rates. On the other hand, they had lower serum albumin, leukocyte count, bicarbonate concentration, and PaO_2_/FiO_2_ ratio. This is a similar trend to that of the hyper‐inflammatory sub‐phenotype identified in clinical studies (Calfee et al., 2014). However, as alluded to, direct comparisons between studies of this nature are challenging and features such as the magnitude of z‐scores and the rank order of variables should be interpreted with caution (Sinha et al., 2021). Advances in methodology alongside more granular phenotyping, using ‐omics technologies, may be required to provide valid comparisons.

This study is exploratory and has important limitations. First, large animals like humans, exhibit variability in their response to injury. This effect of variability can be reduced with the inclusion of a greater number of experimental subjects. This study used a small number of animals which limit the conclusions which can be drawn. However, large animal experimentation, particularly those involving complex critical care interventions, are resource intensive and future studies are likely to face challenges in including substantially greater numbers. Second, although differences were observed between groups and sub‐phenotypes, the duration of the study was short. A longer period of sampling may have captured evolving differences among measured parameters. This may be especially true of indices in which there is likely to be lag after injury, such as renal dysfunction. Third, the choice of clustering technique is flexible, given the variety available to investigators. PAM has several specific disadvantages, not least of which is its vulnerability to the influence of outliers. Future studies may seek to validate clustering solutions by adopting more than one technique (Castela Forte et al., 2019).

In conclusion, we identified preliminary evidence of sub‐phenotypes occurring in animal models of ARDS. These phenotypes are characterized by differences in the severity of shock, systemic inflammation, and lung injury. Sub‐phenotypes bear a qualitative similarity to those identified in clinical cohorts. The method of injury chosen in animal models may tend toward one or the other which should be borne in mind when interpreting preclinical trials of interventions. Further studies are required to confirm these findings and to develop our understanding of the biological underpinning of sub‐phenotypes in ARDS.

## Disclosures

The authors have no conflict of interest to disclose.

## Authors' Contributions

J.E.M. Study conception, model development, study design and conduct, animal surgery, data collection and analyses, and manuscript preparation; K.W. Model development, study design and conduct, animal surgery, data collection and analyses, and manuscript preparation; N.B., M.R.P., and J.Y.S. Model development, study design and conduct, data collection and analyses, and manuscript review; N.G.O., S.P., and S.R. Model development, study design and conduct, animal surgery, and manuscript review; M.B., K.H., M.Y., and K.K.K. Sample analyses, data analyses, data collection, and manuscript review; L.N. Study design, data analyses, and manuscript review; J.K.B. Data analyses and manuscript review; G.L.B. Study design and manuscript review; D.F.M. and J.F.F. Study conception, model development, study design, and manuscript review.

## Supporting information



Fig S1

Fig S2

Fig S3

Fig S4

Data S1
