# Supplementary figures and images for: Characterizing preclinical sub‐phenotypic models of acute respiratory distress syndrome: An experimental ovine study
Source: Physiol Rep. 2021 Oct 7;9(19):e15048. doi: 10.14814/phy2.15048 (PMC8495778; doi:10.14814/phy2.15048)

Plasma cytokines (ng/mL)

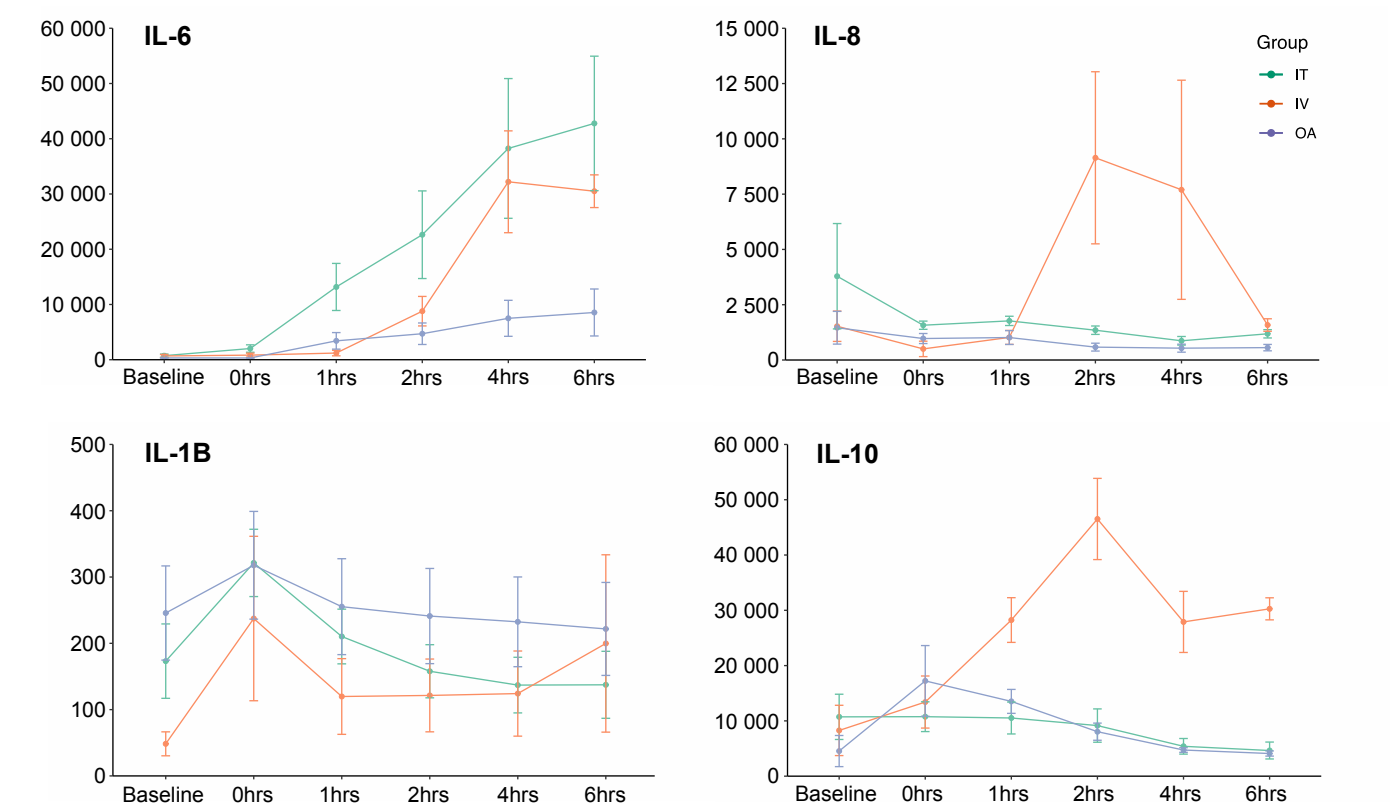

Bronchoalveolar lavage cytokines (ng/mL)

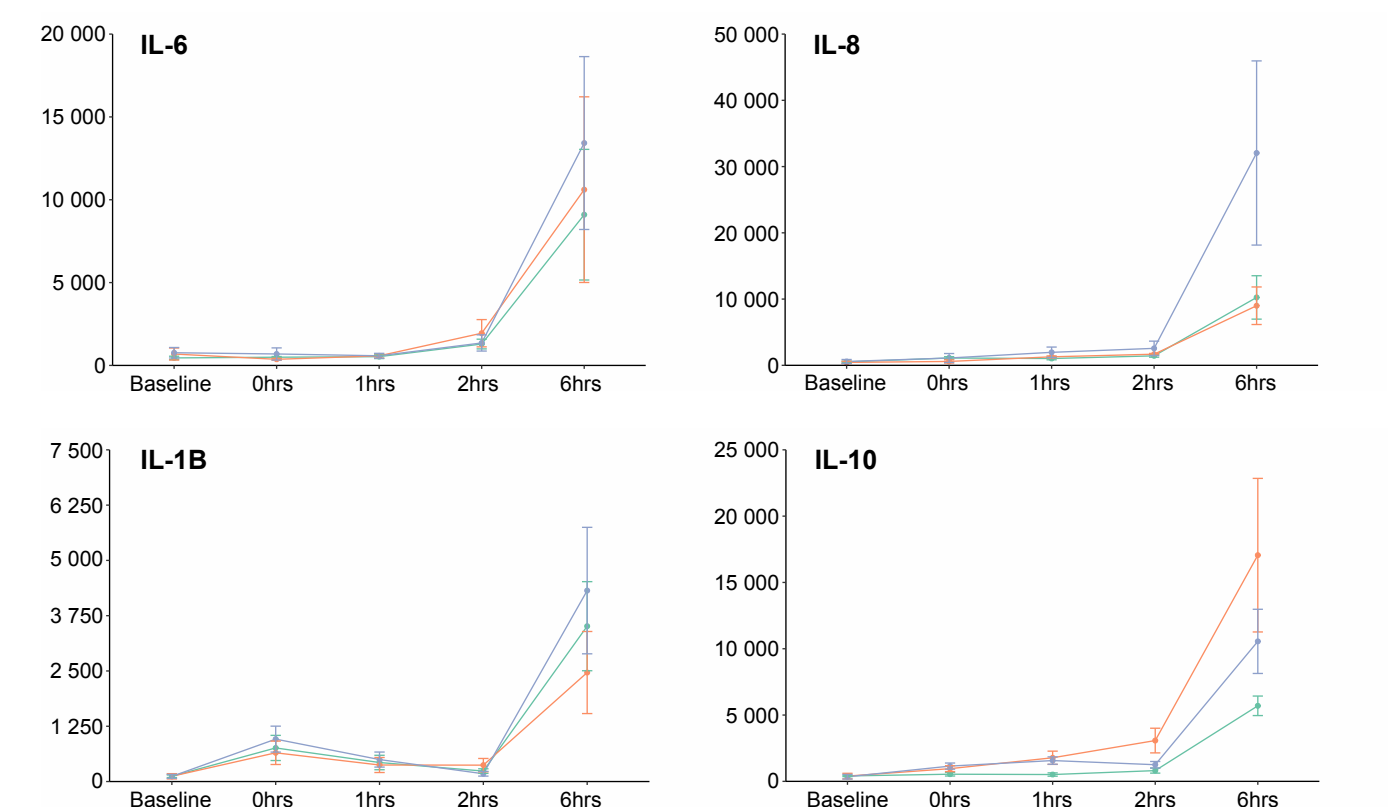

Supplement: Supplementary file 1 — Fig S1 [file PHY2-9-e15048-s004.pdf]

Hemodynamics and acid-base

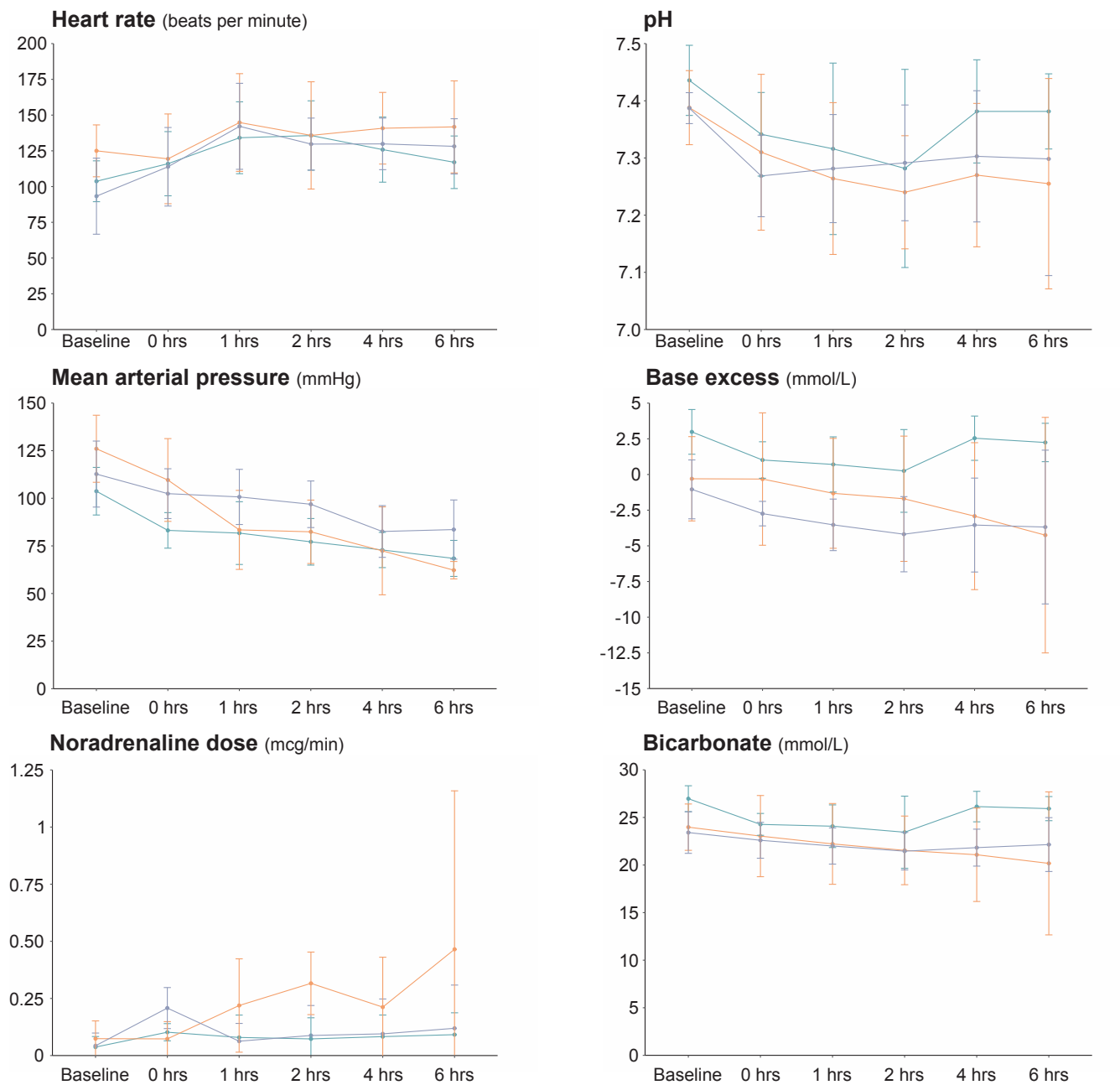

Cumulative urine output and fluid balance

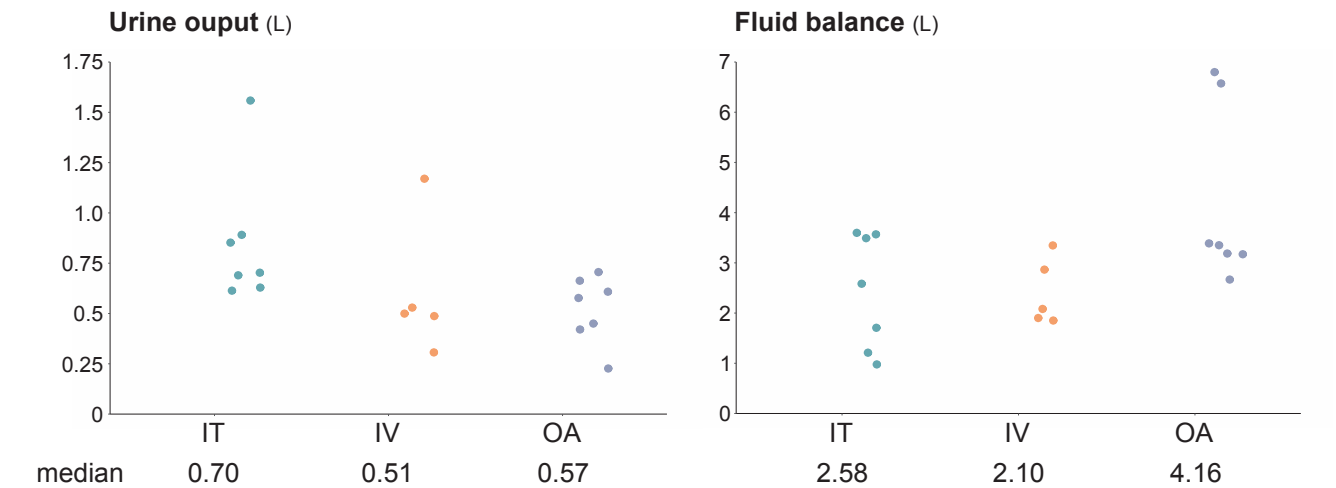

Supplement: Supplementary file 2 — Fig S2 [file PHY2-9-e15048-s002.pdf]

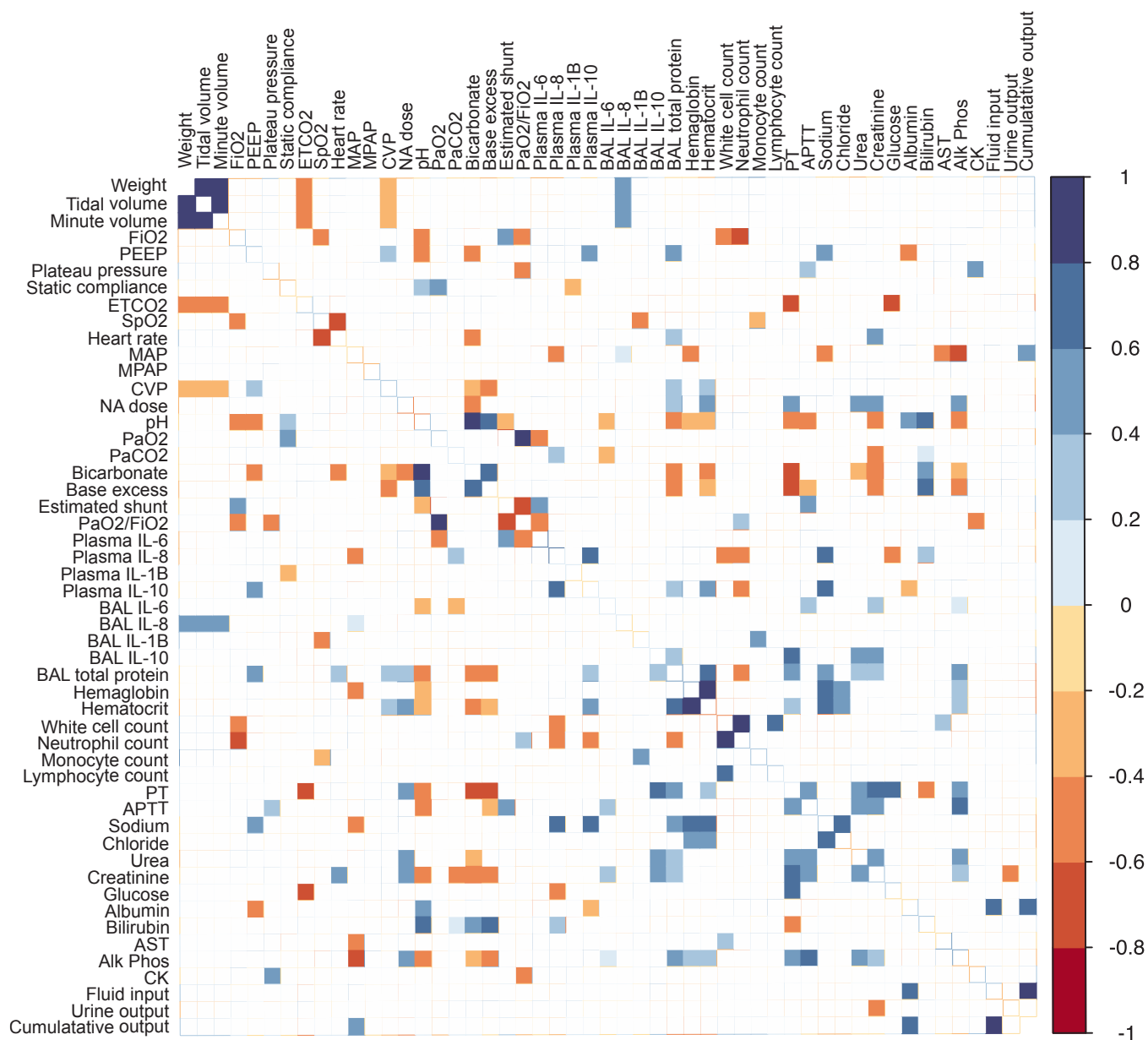

Supplement: Supplementary file 3 — Fig S3 [file PHY2-9-e15048-s005.pdf]
