# Supplementary material for: Characterizing preclinical sub‐phenotypic models of acute respiratory distress syndrome: An experimental ovine study
Source: Physiol Rep. 2021 Oct 7;9(19):e15048. doi: 10.14814/phy2.15048 (PMC8495778; doi:10.14814/phy2.15048)

Top 10 variables in principal componets 1 to 4

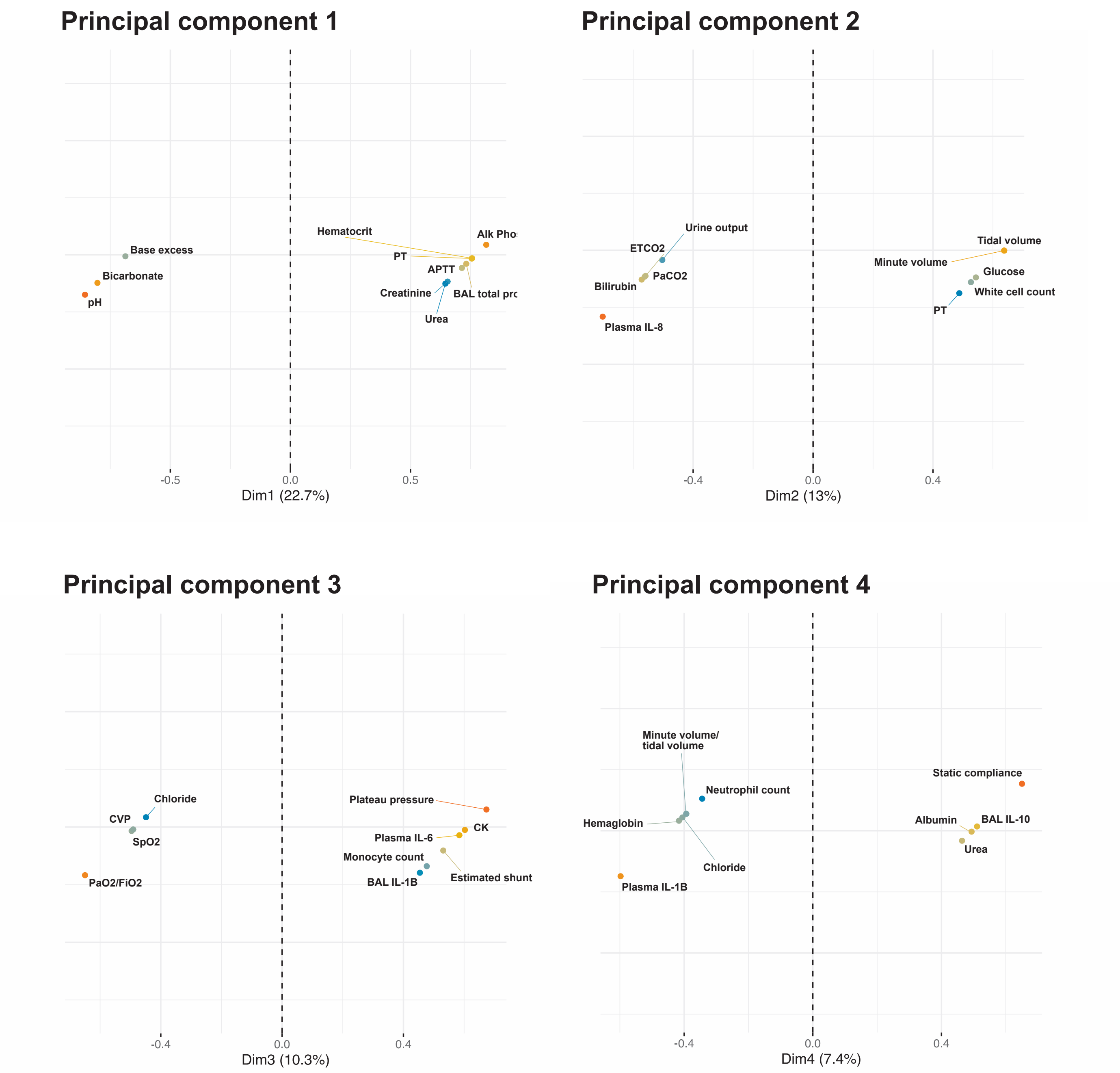

Supplement: Supplementary file 4 — Fig S4 [file PHY2-9-e15048-s001.pdf]
