# Supplementary material for: Characterizing preclinical sub‐phenotypic models of acute respiratory distress syndrome: An experimental ovine study
Source: Physiol Rep. 2021 Oct 7;9(19):e15048. doi: 10.14814/phy2.15048 (PMC8495778; doi:10.14814/phy2.15048)
Supplement: Supplementary file 5 — Data S1 [file PHY2-9-e15048-s003.docx]

**Characterizing pre-clinical sub-phenotypic models of Acute Respiratory Distress Syndrome: an experimental ovine study.**

Jonathan E Millar, Karin Wildi, Nicole Bartnikowski, Mahe Bouquet, Kieran Hyslop, Margaret R Passmore, Katrina K Ki, Nchafatso G Obonyo, Mengyao Yang, Sanne Pedersen, Sacha Rozencwajg, J Kenneth Baillie, Gianluigi Li Blassi, Jacky Y Suen, Daniel F McAuley, John F Fraser.

**Online Supplement**

Contents

A. Sheep preparation

B. Supportive care protocol

C. Model of experimental ARDS

D. Clinical measurements

E. Blood and bronchoalveolar lavage fluid analyses

F. Statistical analysis

G. Results supplement

H. Supplement references

**A. Sheep preparation**

Healthy, farm reared, female sheep (*Ovis aries*, Border Leicester Cross), aged 1-3 years, were used in this study. Animals were allowed a minimum of two weeks acclimatisation at the research facility, during which time they were housed socially in an outdoor enclosure. Prior to experimentation animals underwent veterinary examination and were screened for common ovine pathogens. The day before inclusion, sheep were housed in an indoor pen and given free access to water. Fasting from solid feed was enforced in the 12 hours prior to induction.

Prior to the induction of general anaesthesia, a central venous catheter (Arrow, Teleflex Medical Australia, Mascot, NSW, Australia) was inserted into the right external jugular vein (EJV), and baseline blood samples were obtained. An 8 Fr percutaneous introducer sheath (Arrow, Teleflex Medical Australia, Mascot, NSW, Australia) was also inserted in the right EJV. Animals were then pre-oxygenated for 3 minutes using a facemask. General anaesthesia was induced as described below. Animals were positioned supine. A surgical tracheostomy was performed, and a size 9.0 Portex tracheostomy tube (Smith’s Medical Australia, Sydney, NSW, Australia) was inserted. Correct positioning was confirmed using a flexible video bronchoscope (aScope™, Ambu, Ballerup, Denmark). Thereafter, the tracheostomy was suctioned hourly using an in-line suction catheter.

**B. Supportive care protocol**

Animals were managed throughout the study by experienced critical care practitioners. Expert veterinary advice was available to investigators at all times.

Anaesthesia/analgesia

General anaesthesia was induced using intravenous midazolam (0.5 mg/Kg, Pfizer, Sydney, NSW, Australia) and ketamine (5 mg/kg, Troy Laboratories, Sydney, NSW, Australia). Animals were intubated (size 9.0-10.0 ID Portex endotracheal tube, Smith’s Medical Australia, Sydney, NSW, Australia) and mechanically ventilated. Maintenance of anaesthesia/analgesia was achieved by intravenous infusion of midazolam (0.5-0.8 mg/kg/hr, Pfizer, Sydney, NSW, Australia), ketamine (5-7.5 mg/kg/hr, Troy Laboratories, Sydney, NSW, Australia), and fentanyl (5-10 mcg/kg/hr, Hameln Pharmaceuticals, Hameln, Germany). Total intravenous anaesthesia was maintained until euthanasia.

Once maintenance anaesthesia was established, animals were paralysed with an intravenous bolus of vecuronium (50 mcg/kg, Pfizer, Sydney, NSW, Australia). Adequacy of neuromuscular blockade (NMB) was assessed using the train of four (TOF) response to peripheral nerve stimulation. NMB was maintained through the course of the experiment by an intravenous infusion of vecuronium (50 mcg/kg/hr, Pfizer, Sydney, NSW, Australia).

Mechanical ventilation

Animals were ventilated according to a protocolized lung-protective strategy. In a volume-controlled mode, the ventilator was set to achieve a tidal volume (V_t_) of 6 mL/kg actual body weight (ABW). One of, a Hamilton Galileo (Hamilton Medical, Bonaduz, Switzerland) or a Puritan Bennett 840 (Puritan Bennett, Medtronic, Dublin, Ireland) ventilator was used for the full course of each study. FiO_2_ was adjusted to achieve peripheral oxygen saturations (SpO_2_) in the range 88-93%, hyperoxia was avoided. PEEP was initially set to 5 cmH_2_O and adjusted to maintain a plateau pressure < 30 cmH_2_O. Total PEEP (extrinisic + intrinsic) could not exceed 20 cmH_2_O and could not be reduced < 5 cmH_2_O. Respiratory rate (RR) was adjusted to target a pH 7.30-7.45 but limited to ≤ 35 breaths per minute. An I:E ratio between 1:1 and 1:3 was used throughout.

Fluid and electrolyte management

A maintenance infusion of crystalloid (1-2 mL/kg/hr, compound sodium lactate, Baxter, Sydney, NSW, Australia) was provided for the duration of the study. Fluid therapy was indicated at other times to support haemodynamics, in this case 100 mL boluses of compound sodium lactate were titrated to effect.

Electrolyte levels were assessed 4-hourly. Serum potassium levels were maintained > 3.5 mmol/L by i.v. infusion of 10-20 mmol potassium chloride (AstraZeneca, Sydney, NSW, Australia). All large volume gastric losses were returned via the orogastric tube.

Monitoring and haemodynamic management

All animals were monitored according to the ‘*Recommendations for standards of monitoring during anaesthesia and recovery 2015: Association of Anaesthetists of Great Britain and Ireland*’ (1), including; pulse oximetry, 3-lead electrocardiogram, and continuous waveform capnography. In addition, the central venous catheter (CVC) was transduced as a measure of central venous pressure.

Invasive arterial blood pressure monitoring was established by cannulation of the left facial artery (20G Leadercath arterial, Vygon, Écouen, France). Using the 8 Fr percutaneous sheath introducer positioned in the right EJV, a pulmonary artery catheter (PAC) was inserted (7.5 Fr Swan-Ganz CCOmbo, Edwards Life Sciences, Irvine, CA, USA). Positioning was confirmed by obtaining a satisfactory waveform. Continuous cardiac output monitoring (Vigilance monitor, Edwards Lifesciences, Irvine, CA, USA) was instituted, with cardiac index estimated by; cardiac output/BSA [weight (kg)^0.67^ x 0.0842]. A 14 Fr urinary catheter (Gildana Healthcare, Oakleigh, VIC, Australia) was inserted and connected to an hourly urometer to allow for accurate quantification of urine output. A 16 Fr orogastric tube (ConvaTec, Glen Waverley, VIC, Australia) was inserted and left on free drainage.

A mean arterial pressure ≥ 65 mmHg was targeted. In the face of sustained hypotension, repeated 100 mL boluses of compound sodium lactate (Baxter, Sydney, NSW, Australia) were given until; CVP ≥ 8 mmHg and/or the animal was no longer fluid responsive (failure to increase stroke volume > 10% by PAC). Thereafter, noradrenaline (80 mcg/mL in 5% dextrose, Hospira, Lake Forrest, IL, USA) was commenced at 80 mcg/min and the dose titrated at 5-minute intervals.

Euthanasia

At the end of each study, animals were euthanised by i.v. injection of phenobarbitone (142.5 mg/kg, Aspen Pharma, Dandenong, NSW, Australia). After death was confirmed (absence of cardiac electrical activity, blood pressure, and cardiac output monitoring), organs were retrieved surgically. Animal carcasses were stored and subsequently disposed of by incineration.

**C.** **Model of experimental ARDS**

After instrumentation, animals were injured in the supine position.

Oleic acid preparation

A total dose of 0.06 ml/kg OA was used. Firstly, 0.03 mL/kg OA (O1008, Sigma-Aldrich, Castle Hill, NSW, Australia) was suspended in 20 mL arterial blood and 150 IU porcine heparin (Pfizer, Sydney, NSW, Australia). This mixture was administered via the distal port of the right EJV central venous catheter, followed by a flush of 50 mL 0.9% sodium chloride (Baxter, Sydney, NSW, Australia). The animal could recover and after 15 minutes this procedure was repeated. When 15 minutes elapsed from the second dose, arterial blood gas analysis, at a minimum PEEP of 5 cmH_2_O, was used to confirm a PaO_2_/FiO_2_ ratio <150 mmHg.

*E. coli* lipopolysaccharide preparation

Immediately after a PaO_2_/FiO_2_ ratio <150 mmHg was confirmed, *E. coli* LPS was administered via one of two routes to animals assigned to these groups.

For animals assigned to the i.t. LPS group, 50 mcg *E. coli* LPS (O55:B5, Sigma-Aldrich, Castle Hill, NSW, Australia), diluted in 10 mL 0.9% sodium chloride (Baxter, Sydney, NSW, Australia), was administered, via a designated video bronchoscope, to each main bronchus.

For animals assigned to the i.v. LPS group, 60 mcg *E. coli* LPS (O55:B5, Sigma-Aldrich, Castle Hill, NSW, Australia) diluted in 50 mL 0.9% sodium chloride (Baxter, Sydney, NSW, Australia), was administered infused via the CVC at a rate of 1 mcg/kg/hr for one hour.

**D. Clinical measurements**

Hemodynamic and ventilatory data (including data derived from the PAC) were continuously monitored and automatically recorded at 5-minute intervals using a data monitoring system (Solar 8000, GE Healthcare, Waukesha, WI, USA) coupled with custom software. Urine and oro-gastric outputs were recorded on a pre-piloted observation chart on an hourly basis.

**E.** **Blood and bronchoalveolar lavage fluid analyses**

Blood

Whole blood was sampled from the facial artery catheter. Arterial blood gas analysis was undertaken on at least an hourly basis (ABL800 FLEX, Radiometer, Copenhagen, Denmark). At baseline, zero hours (injury), 1 hour, 2 hours, 4 hours, and 6 hours, blood was sampled for routine laboratory haematological and biochemical testing. Testing was undertaken by an independent veterinary laboratory (IDEXX Laboratories, Brisbane, Australia) to clinical standards. Blood was also sampled for plasma cytokine measurements. The concentration of IL-6, IL-1β, IL-8 and IL-10 in plasma and bronchoalveolar lavage (BAL) fluid was quantified by in-house ELISAs. Positive internal controls were used to ensure that inter- and intra- plate variability was < 10% and confirm the precision and accuracy of all ELISA assays.

Bronchoalveolar lavage fluid

BAL was undertaken by an experienced bronchoscopist using a video bronchoscope (aScope™, Ambu, Ballerup, Denmark). At each examination the right and left middle and lower lobes were sampled. Each lobe was injected with 20 mL sterile 0.9% sodium chloride (Baxter, Sydney, NSW, Australia) and gentle suction was applied. The lavage fluid was collected in a sterile universal container. BAL fluid was centrifuged, and the supernatant collected for ELISA analysis. BAL total protein content was measured using a BCA (bicinchoninic acid) protein assay kit (Pierce BCA protein assay, ThermoFisher, VIC, Australia).

**F. Statistical analysis**

The following R packages were used in the analysis, all versions as of 2020-11-04: tidyverse; psych; FactoMineR; factoextra; lme4; missRanger; rstatix; cluster; corrplot; RColorBrewer; cowplot; naniar; gghalves; NbClust; fpc; officer; flextable; gtsummary.

**G. Results supplement**

**Supplementary Table E1.** Hematological and biochemical characteristics at zero hours (injury). Data are presented as median (interquartile range).

|  | **Overall** (n=19) | **OA** (n=7) | **IT** (n=7) | **IV** (n=5) |
| --- | --- | --- | --- | --- |
| **Haemaglobin** (g/L) | 107 (94-114) | 109 (88-114) | 110 (107-115) | 95 (93-106) |
| **Haematocrit** | 0.32 (0.3-0.34) | 0.31 (0.3-0.32) | 0.34 (0.32-0.36) | 0.31 (0.29-0.32) |
| **White cell count** (x 10^^9^/L) | 2 (1.5-2.3) | 2.2 (2.1-2.4) | 1.7 (1.4-2) | 1.7 (1.4-3.2) |
| **Neutrophil count** (x 10^^9^/L) | 0.4 (0.2-0.7) | 0.7 (0.4-0.9) | 0.3 (0.2-0.4) | 0.3 (0.3-0.7) |
| **Monocyte count** (x 10^^9^/L) | 0 (0-0.1) | 0 (0-0.2) | 0 (0-0) | 0.1 (0.1-0.1) |
| **Lymphocyte count** (x 10^^9^/L) | 1.4 (1.1-1.7) | 1.5 (1.2-1.8) | 1.3 (1.1-1.5) | 1.4 (1-2.3) |
| **PT** (s) | 14.5 (13.8-15.7) | 15.8 (14.7-16.1) | 14.2 (13.8-14.4) | 14.4 (13.3-15.3) |
| **APTT** (s) | 30 (27-36.5) | 31 (28-36) | 27 (27-31) | 32 (28-42) |
| **Sodium** (mmol/L) | 141 (140-142) | 140 (140-141) | 142 (140-142) | 142 (141-143) |
| **Chloride** (mmol/L) | 105 (103-106) | 105 (104-107) | 105 (103-106) | 104 (100-105) |
| **Urea** (mmol/L) | 5.7 (4.8-7) | 5.5 (4.6-6.3) | 5.5 (5.2-7) | 7 (6.7-7.5) |
| **Creatinine** (mmol/L) | 0.07 (0.06-0.07) | 0.07 (0.07-0.08) | 0.06 (0.06-0.06) | 0.07 (0.07-0.07) |
| **Glucose** (mmol/L) | 5.7 (4-7.3) | 6.4 (5.9-9.5) | 4 (3.7-4.3) | 7.3 (4.1-8) |
| **Albumin** (g/L) | 26 (24-28) | 28 (26-30) | 25 (24-25) | 27 (24-27) |
| **Bilirubin** (μmol/L) | 2 (1-2.8) | 2 (1.5-4) | 2 (1.3-2) | 1 (1-1) |
| **AST** (IU/L) | 87 (77-113) | 86 (78-102) | 94 (77-122) | 88 (81-103) |
| **ALP** (IU/L) | 124 (94-160) | 95 (83-153) | 124 (92-132) | 186 (115-207) |
| **CK** (IU/L) | 233 (178-323) | 215 (187-273) | 347 (240-446) | 212 (136-250) |

**Supplementary Table E2.** Hematological and biochemical characteristics at 6 hours. Data are presented as median (interquartile range).

|  | **Overall** (n=19) | **OA** (n=7) | **IT** (n=7) | **IV** (n=5) |
| --- | --- | --- | --- | --- |
| **Haemaglobin** (g/L) | 100 (91-110) | 98 (91-109) | 94 (90-101) | 109 (105-114) |
| **Haematocrit** | 0.3 (0.27-0.35) | 0.28 (0.28-0.32) | 0.28 (0.26-0.31) | 0.34 (0.33-0.38) |
| **White cell count** (x 10^^9^/L) | 2.2 (1.5-2.7) | 2.7 (2.2-3.8) | 2.1 (1.5-2.5) | 1.6 (1.1-2.2) |
| **Neutrophil count** (x 10^^9^/L) | 1.3 (0.5-1.6) | 1.4 (1.4-2.7) | 1.2 (0.9-1.5) | 0.4 (0.3-0.5) |
| **Monocyte count** (x 10^^9^/L) | 0.1 (0.1-0.2) | 0.1 (0.1-0.2) | 0.1 (0-0.2) | 0.1 (0.1-0.2) |
| **Lymphocyte count** (x 10^^9^/L) | 0.8 (0.7-1.1) | 0.8 (0.8-1.1) | 0.8 (0.7-0.9) | 1.1 (0.7-1.5) |
| **PT** (s) | 17.6 (16.1-19.3) | 17.6 (17.2-18.5) | 15.2 (14.8-17) | 19.3 (19.3-21.2) |
| **APTT** (s) | 34.7 (29-37) | 33 (29-35) | 30 (29.5-38.5) | 37 (37-37) |
| **Sodium** (mmol/L) | 143 (140-144) | 142 (140-143) | 143 (140-143) | 144 (144-147) |
| **Chloride** (mmol/L) | 108 (105-109) | 107 (106-110) | 107 (105-108) | 109 (109-112) |
| **Urea** (mmol/L) | 5 (4.3-6.9) | 4.6 (4.3-5.2) | 5.2 (4.9-6.3) | 8 (3.9-8.2) |
| **Creatinine** (mmol/L) | 0.08 (0.07-0.09) | 0.08 (0.07-0.08) | 0.08 (0.06-0.08) | 0.09 (0.07-0.1) |
| **Glucose** (mmol/L) | 1.8 (1.4-2.8) | 2.7 (2.1-3.5) | 1.8 (1.3-2.2) | 1.5 (1.1-2.5) |
| **Albumin** (g/L) | 21 (19-24) | 25 (22-26) | 20 (20-21) | 19 (16-22) |
| **Bilirubin** (μmol/L) | 3 (2-5.5) | 3 (1.5-3) | 4 (2-9) | 2 (1-7) |
| **AST** (IU/L) | 122 (94-137) | 94 (87-115) | 126 (108-154) | 138 (128-201) |
| **ALP** (IU/L) | 101 (63-134) | 75 (56-101) | 101 (58-116) | 134 (133-220) |
| **CK** (IU/L) | 368 (261-509) | 305 (272-479) | 503 (378-601) | 357 (241-368) |

**Supplementary Table E3.** Linear mixed model group:time interactions.

| **Variable** | **Group:time P value** | **Sig. adj. post-hoc** |
| --- | --- | --- |
| FiO2 | 0.093 |  |
| PEEP | 0.637 |  |
| Plateau pressure | 0.744 |  |
| Static compliance | 0.022 | No |
| ETCO2 | 0.720 |  |
| SpO2 | 0.992 |  |
| Heart rate | 0.709 |  |
| MAP | 0.001 | Yes |
| MPAP | 0.532 |  |
| CVP | 0.627 |  |
| Noradrenaline dose | <0.001 | Yes |
| pH | 0.381 |  |
| PaO2 | 0.877 |  |
| PaCO2 | 0.344 |  |
| Bicarbonate | 0.184 |  |
| Base excess | 0.265 |  |
| Estimated shunt | 0.688 |  |
| PaO2/FiO2 | 0.727 |  |
| IL6 plasma | <0.001 | Yes |
| IL8 plasma | <0.001 | Yes |
| IL1B plasma | 0.053 |  |
| IL10 plasma | <0.001 | Yes |
| IL6 BAL | 0.986 |  |
| IL8 BAL | 0.031 | No |
| IL1B BAL | 0.748 |  |
| IL10 BAL | 0.013 | Yes |
| Hemaglobin | 0.017 | No |
| Hematocrit | 0.018 | No |
| White cell count | <0.001 | Yes |
| Neutrophil count | <0.001 | Yes |
| Lymphocyte count | 0.023 | No |
| Monocyte count | 0.020 | No |
| PT | 0.021 | No |
| APTT | 0.342 |  |
| Sodium | 0.003 | Yes |
| Chloride | 0.094 |  |
| Urea | 0.754 |  |
| Creatinine | 0.744 |  |
| Glucose | 0.592 |  |
| Albumin | 0.133 |  |
| Bilirubin | 0.002 | Yes |
| AST | <0.001 | No |
| Alk Phos | 0.583 |  |
| CK | 0.377 |  |

**Supplementary Table E4.** Post-hoc comparisons.

Tukey’s honest significant differences test. P values adjusted using false discovery set at 5%.

Mean arterial pressure (MAP), mmHg

| **Timepoint** | **Group 1** | **Group 2** | **Mean difference** | **95% CIs** | **adj. P value** |
| --- | --- | --- | --- | --- | --- |
| 0 hrs | IT | IV | 27 | 7 – 47 | 0.012 |
| 0 hrs | IT | OA | 19 | 0 – 38 | 0.046 |
| 2 hrs | IT | OA | 20 | 1 – 38 | 0.034 |
| 6 hrs | IV | OA | 21 | 1 – 42 | 0.040 |

Noradrenaline dose, mcg/kg/min

| **Timepoint** | **Group 1** | **Group 2** | **Mean difference** | **95% CIs** | **adj. P value** |
| --- | --- | --- | --- | --- | --- |
| 0 hrs | IT | OA | 0.211 | 0.015 – 0.408 | 0.034 |
| 0 hrs | IV | OA | 0.270 | 0.054 – 0.485 | 0.014 |
| 2 hrs | IT | IV | 0.486 | 0.125 – 0.848 | 0.008 |
| 2 hrs | IV | OA | -0.456 | -0.818 – -0.095 | 0.013 |

Plasma IL-6, ng/mL

| **Timepoint** | **Group 1** | **Group 2** | **Mean difference** | **95% CIs** | **adj. P value** |
| --- | --- | --- | --- | --- | --- |
| 0 hrs | IT | OA | -1708 | -3306 – -109 | 0.036 |
| 1 hrs | IT | IV | -11964 | -23051 – -877 | 0.034 |
| 6 hrs | IT | OA | -21952 | -63392 – -5062 | 0.021 |

Plasma IL-8, ng/mL

| **Timepoint** | **Group 1** | **Group 2** | **Mean difference** | **95% CIs** | **adj. P value** |
| --- | --- | --- | --- | --- | --- |
| 0 hrs | IT | IV | -1072 | -2000 – -144 | 0.023 |
| 2 hrs | IT | IV | 7796 | 1192 – 14400 | 0.020 |
| 2 hrs | IV | OA | -8561 | -15165 – -1957 | 0.011 |
| 6 hrs | IV | OA | -1022 | -1733 – -270 | 0.008 |

Plasma IL-10, ng/mL

| **Timepoint** | **Group 1** | **Group 2** | **Mean difference** | **95% CIs** | **adj. P value** |
| --- | --- | --- | --- | --- | --- |
| 1 hrs | IT | IV | 17717 | 6547 – 28887 | 0.002 |
| 1 hrs | IV | OA | -14715 | -25885 – -3545 | 0.010 |
| 2 hrs | IT | IV | 37367 | 22441 – 52294 | <0.001 |
| 2 hrs | IV | OA | -38473 | -53400 – -23547 | <0.001 |
| 4 hrs | IT | IV | 22499 | 12493 – 32505 | <0.001 |
| 4 hrs | IV | OA | -23171 | -33177 – -13165 | <0.001 |
| 6 hrs | IT | IV | 25626 | 20461 – 30792 | <0.001 |
| 6 hrs | IV | OA | -26170 | -31335 – 21004 | <0.001 |

BAL IL-10, ng/mL

| **Timepoint** | **Group 1** | **Group 2** | **Mean difference** | **95% CIs** | **adj. P value** |
| --- | --- | --- | --- | --- | --- |
| 1 hrs | IT | IV | 1264 | 138 – 2390 | 0.027 |
| 1 hrs | IT | OA | 1053 | 25 – 2081 | 0.044 |
| 2 hrs | IT | IV | 2267 | 365 – 4168 | 0.019 |

White cell count, x10^9/L

| **Timepoint** | **Group 1** | **Group 2** | **Mean difference** | **95% CIs** | **adj. P value** |
| --- | --- | --- | --- | --- | --- |
| 4 hrs | IV | OA | 1.24 | 0 – 2.5 | 0.049 |

Neutrophil count, x10^9/L

| **Timepoint** | **Group 1** | **Group 2** | **Mean difference** | **95% CIs** | **adj. P value** |
| --- | --- | --- | --- | --- | --- |
| 4 hrs | IV | OA | 0.97 | 0 – 1.9 | 0.034 |

Sodium, mmol/L

| **Timepoint** | **Group 1** | **Group 2** | **Mean difference** | **95% CIs** | **adj. P value** |
| --- | --- | --- | --- | --- | --- |
| 1 hrs | IV | OA | -3 | -6 – 0 | 0.024 |
| 6 hrs | IT | IV | 4 | 1 – 7 | 0.008 |
| 6 hrs | IV | OA | -4 | -7 – -1 | 0.005 |

Bilirubin, umol/L

| **Timepoint** | **Group 1** | **Group 2** | **Mean difference** | **95% CIs** | **adj. P value** |
| --- | --- | --- | --- | --- | --- |
| 4 hrs | IT | OA | -2 | -4 – 0 | 0.042 |

**Supplementary Table E5.** Data missingness.

Variables with > 2 cases missing.

| **Variable** | **Number**  **missing** | **Non-imputed**  Median (IQR) | **Imputed**  Median (IQR) |
| --- | --- | --- | --- |
| BAL IL-8 6 hrs | 5 | 12060 (5482 – 16086) | 12108 (2724 – 20484) |
| PaCO2 6 hrs | 4 | 52 (42 – 68) | 54 (42 – 68) |
| BAL IL-10 6 hrs | 4 | 7884 (5767 – 16643) | 10064 (6175 – 24086) |
| ETCO2 6 hrs | 3 | 34 (31 – 38) | 34 (31 – 39) |
| CVP 6hrs | 3 | 15 (14 – 17) | 15 (14 – 16) |
| EF 6 hrs | 3 | 42 (37 – 51) | 44 (39 – 53) |
| BAL IL-6 6 hrs | 3 | 9322 (2786 – 12846) | 9956 (2573 – 12907) |

**Supplementary Table E6.** Measures of optimal cluster number

| **Measure** | **Number of clusters** |
| --- | --- |
| Hubert | 0 |
| Dindex (Lebart) | 0 |
| Frey | 1 |
| Cindex (Hubert and Levin) | 2 |
| Duda | 2 |
| PseudoT2 (Duda and Hart) | 2 |
| Beale | 2 |
| Ratkowsky | 2 |
| Marriot | 3 |
| TrCovW (Milligan and Cooper) | 3 |
| Ball | 3 |
| TraceW (Milligan and Cooper) | 4 |
| Scott | 5 |
| Silhouette | 6 |
| PtBiserial (Milligan) | 6 |
| Dunn | 6 |
| Hartigan | 7 |
| SDindex (Halkidi) | 8 |
| DB (Davies and Bouldin) | 9 |
| McClain | 9 |
| Rubin | 16 |
| KL (Krzanowski and Lai) | 17 |
| CH (Calinski and Harabasz) | 17 |
| CCC (Sarie) | 17 |
| Friedman | 18 |
| SDbw (Halkidi and Vazirgiannis) | 18 |

**Supplementary Figure E1.** Plasma and bronchoalveolar lavage cytokine concentrations.

Data are presented as mean and 95% confidence intervals.

**Supplementary Figure E2.** Hemodynamics, acid-base, and fluid balance.

Data are presented as mean and 95% confidence intervals.

**Supplementary Figure E3.** Correlation of variables.

Spearman’s correlation. Pair-wise correlations with a p value > 0.05 are omitted.

**Supplementary Figure E4.** Contribution of variables to principal components 1-4.

The ‘top 10’ variables describing each principal component are shown.

**H. Supplement references**

1. Checketts MR, Alladi R, Ferguson K, Gemmell L, Handy JM, Klein AA, Love NJ, Misra U, Morris C, Nathanson MH, Rodney GE, Verma R, Pandit JJ, Association of Anaesthetists of Great B, Ireland. Recommendations for standards of monitoring during anaesthesia and recovery 2015: Association of Anaesthetists of Great Britain and Ireland. *Anaesthesia* 2016; 71: 85-93.

2. Matute-Bello G, Downey G, Moore BB, Groshong SD, Matthay MA, Slutsky AS, Kuebler WM, Acute Lung Injury in Animals Study G. An official American Thoracic Society workshop report: features and measurements of experimental acute lung injury in animals. *Am J Respir Cell Mol Biol* 2011; 44: 725-738.
